# Supplementary material for: BES1 is activated by EMS1-TPD1-SERK1/2-mediated signaling to control tapetum development in Arabidopsis thaliana
Source: Nat Commun. 2019 Sep 13;10:4164. doi: 10.1038/s41467-019-12118-4 (PMC6744560; doi:10.1038/s41467-019-12118-4)
Supplement: Supplementary file 1 — Supplementary Information [file 41467_2019_12118_MOESM1_ESM.pdf]

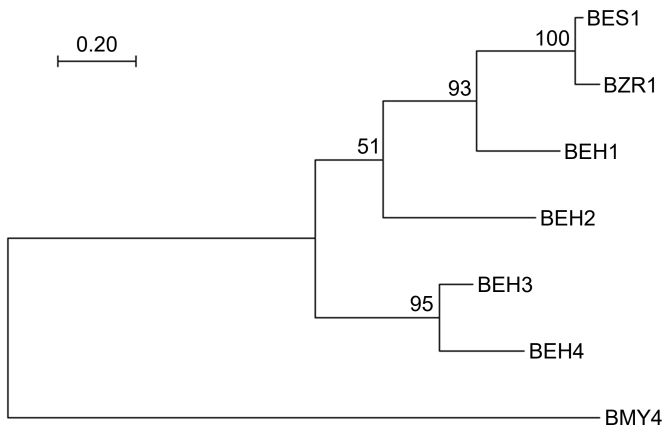

Supplementary Figure 1. Phylogenetic analysis of the BES1 family members. Neighbor-joining tree was generated using the Jones-Taylor-Thornton (JTT) model and gamma-distributed (gamma = 0.97) with 1,000 bootstrap replicates. Bootstrap values are shown along the branches. Scale bar indicates amino acid substitutions per site. BMY4 (BETA-AMYLASE 4) was used as an outgroup control due to its relatively higher score in a WU-BLAST search (https://www.arabidopsis.org/wublast/index2.jsp) using full-length amino acid sequence of BES1.

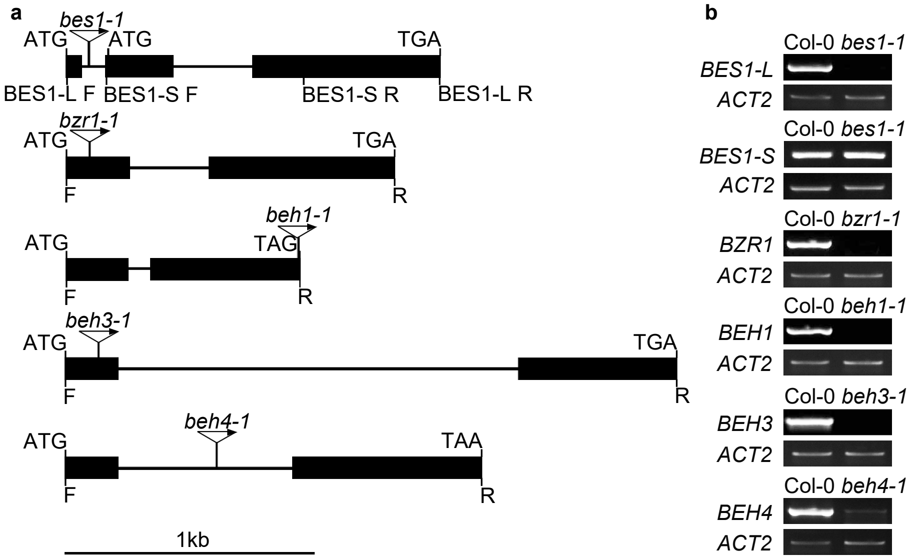

Supplementary Figure 2. T-DNA insertion sites and expression levels of *BES1*, *BZR1*, *BEH1*, *BEH3*, and *BEH4* in their corresponding single mutants. **a** T-DNA insertion site for each of the five genes. **b** RT-PCR results confirming no full-length *BES1*, *BZR1*, *BEH1*, and *BEH3* expression in the corresponding single mutant. *beh4-1* is a knockdown mutant.

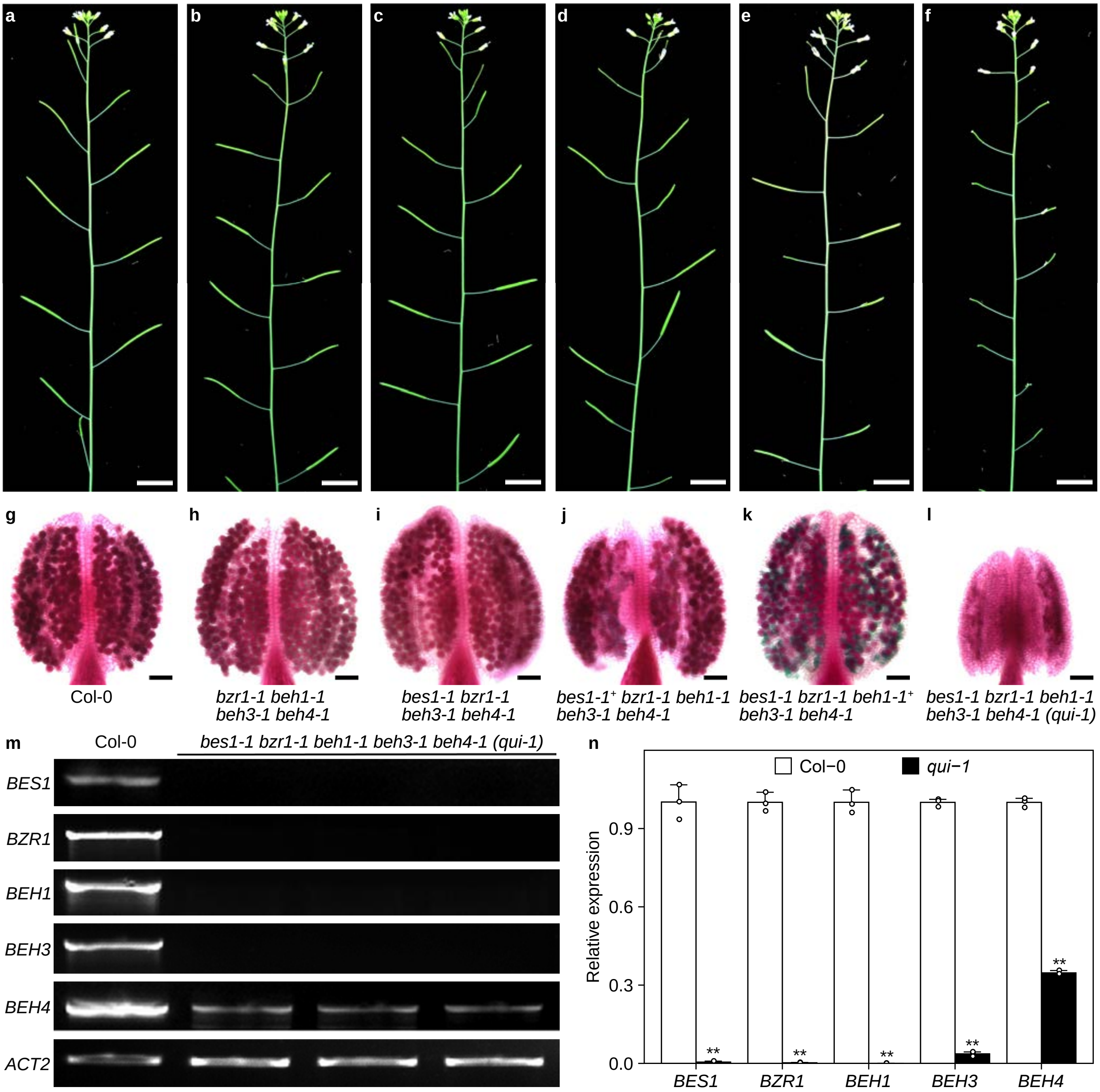

Supplementary Figure 3. Quintuple mutant but not the quadruple mutants generated from the T-DNA insertion lines show a complete male sterility phenotype. **a-f** Inflorescence phenotypes of Col-0 (**a**) and various mutants (**b-f**). Quadruple mutants *bzr1-1 beh1-1 beh3-1 beh4-1* and *bzr1-1 bes1-1 beh3-1 beh4-1* are fertile (**b, c**). *bes1-1⁺ bzr1-1 beh1-1 beh3-1 beh4-1* (**d**) and *beh1-1⁺ bes1-1 bzr1-1 beh3-1 beh4-1* (**e**) mutants show partial sterility, and homozygous quintuple mutant (*qui-1*) is completely sterile (**f**). **g-l** Alexander stained anthers from the plants corresponding to (**a-f**), respectively. **m-n** Quantitative RT-PCR (**m**) and RT-PCR (**n**) analyses to confirm the *qui-1* mutant. Data in (**n**) are presented as mean and s.d. (n = 3). Asterisks indicate significant difference ($P < 0.01$, two-tailed t-test). Scale bars represent 1 cm in **a-f**, 50 μm in **g-l**.

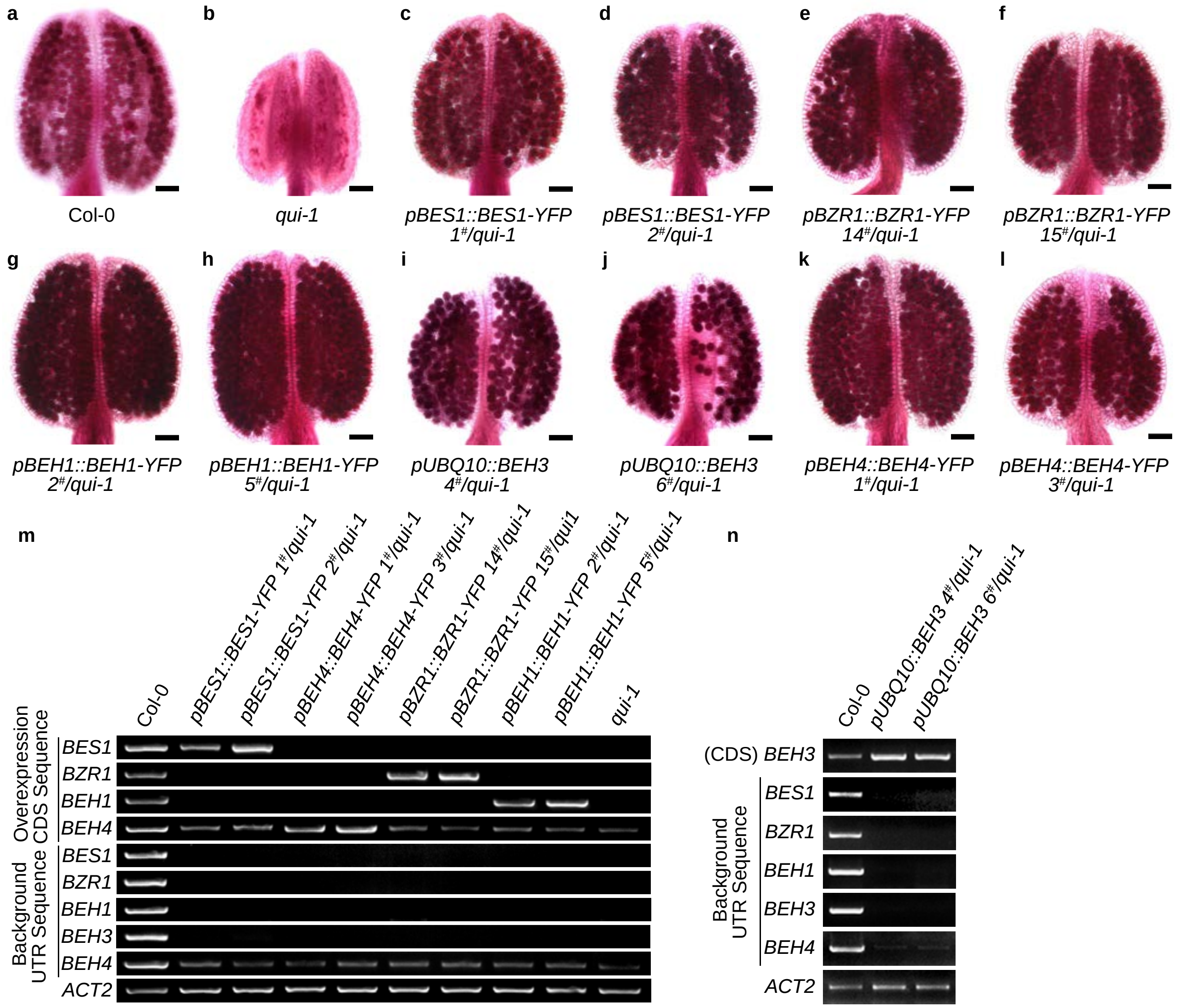

Supplementary Figure 4. Expression of *BES1* family members can fully complement pollen developmental defects in *qui-1*. **a-l** Alexander stained anthers of Col-0 (**a**), *qui-1* (**b**), independent lines of *qui-1* transformed with *pBES1::BES1-YFP* (**c**, **d**), *pBZR1::BZR1-YFP* (**e**, **f**), *pBEH1::BEH1-YFP* (**g**, **h**), *pUBQ10::BEH3-FLAG* (**i**, **j**), and *pBEH4::BEH4-YFP* (**k**, **l**). Scale bars represent 50 μm. **m-n** Confirmation of the *qui-1* background and transgene expression of the plants corresponding to **a-l**. CDS RT-PCR results indicating the expression of the transgenes. UTR RT-PCR results confirm the *qui-1* background.

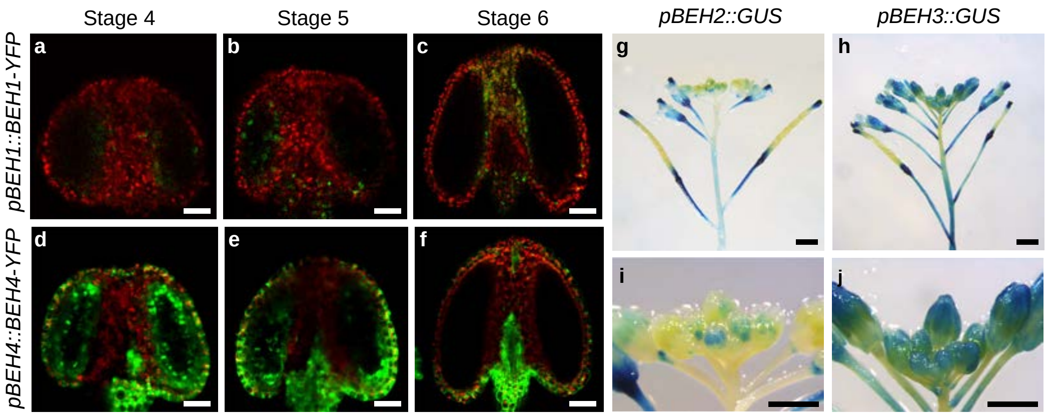

Supplementary Figure 5. BEH1 and BEH4, or BEH2 and BEH3 can be detected in anthers or in inflorescences. **a-c** BEH1-YFP signals were detected in the anthers from stages 4 to 6. **d-f** BEH4-YFP signals were found in the anthers from stage 4 to 6. **g**, **i** GUS staining of an inflorescence of *pBEH2::GUS*. **h**, **j** GUS staining of an inflorescence of *pBEH3::GUS*. Scale bars represent 20 μm in **a-f** and 1 mm in **g-j**.

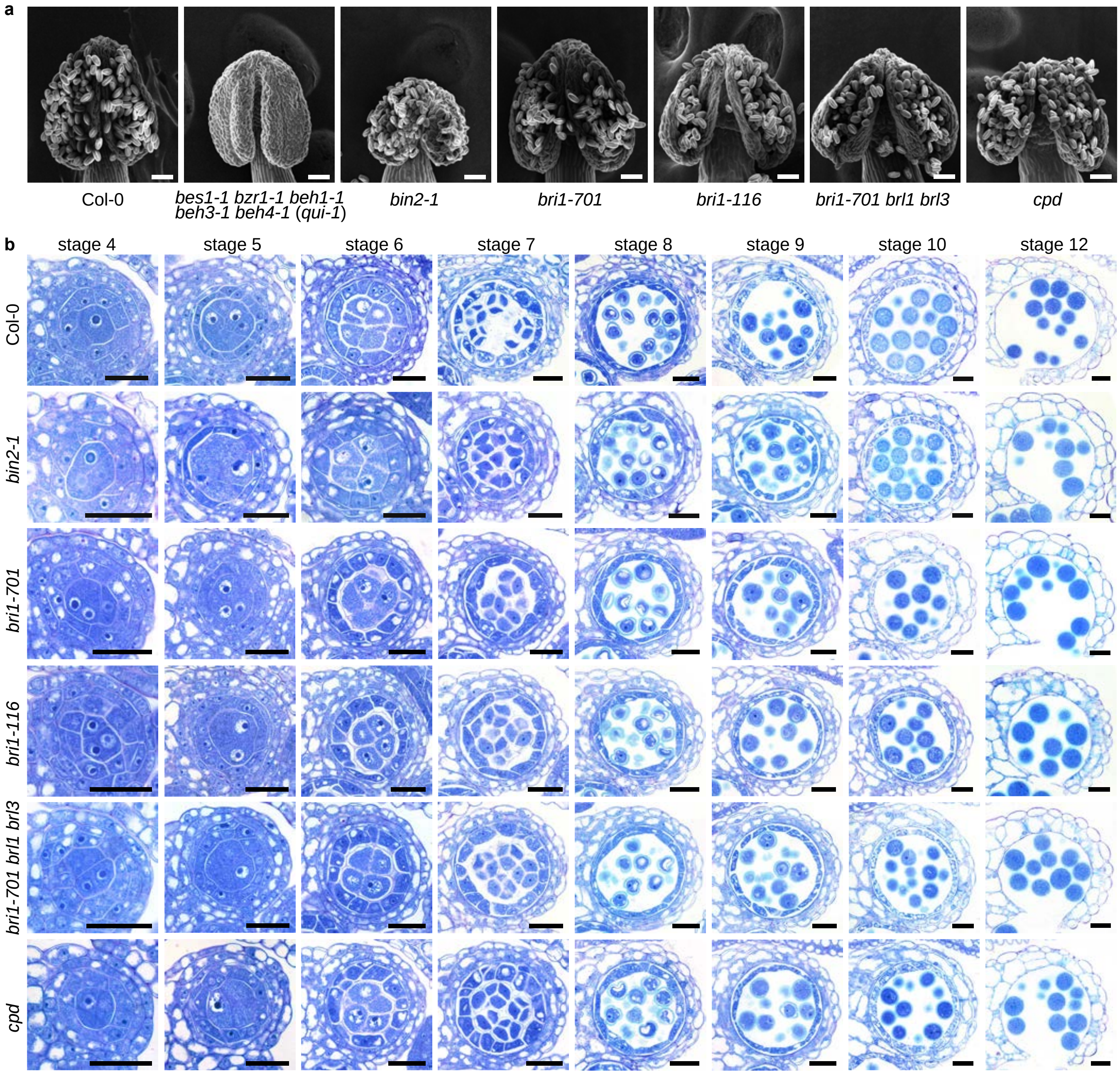

Supplementary Figure 6. BR signaling and biosynthetic mutants are able to produce pollens in their anthers. **a** Scanning electronic microscopic images of anthers from Col-0, *qui-1*, and various BR signaling and biosynthetic mutants. **b** Toluidine blue stained anther semithin sections to show the development of anthers of various BR signaling and biosynthetic mutants from stages 4-12. Scale bars represent 50 nm in **a** and 20 nm in **b**.

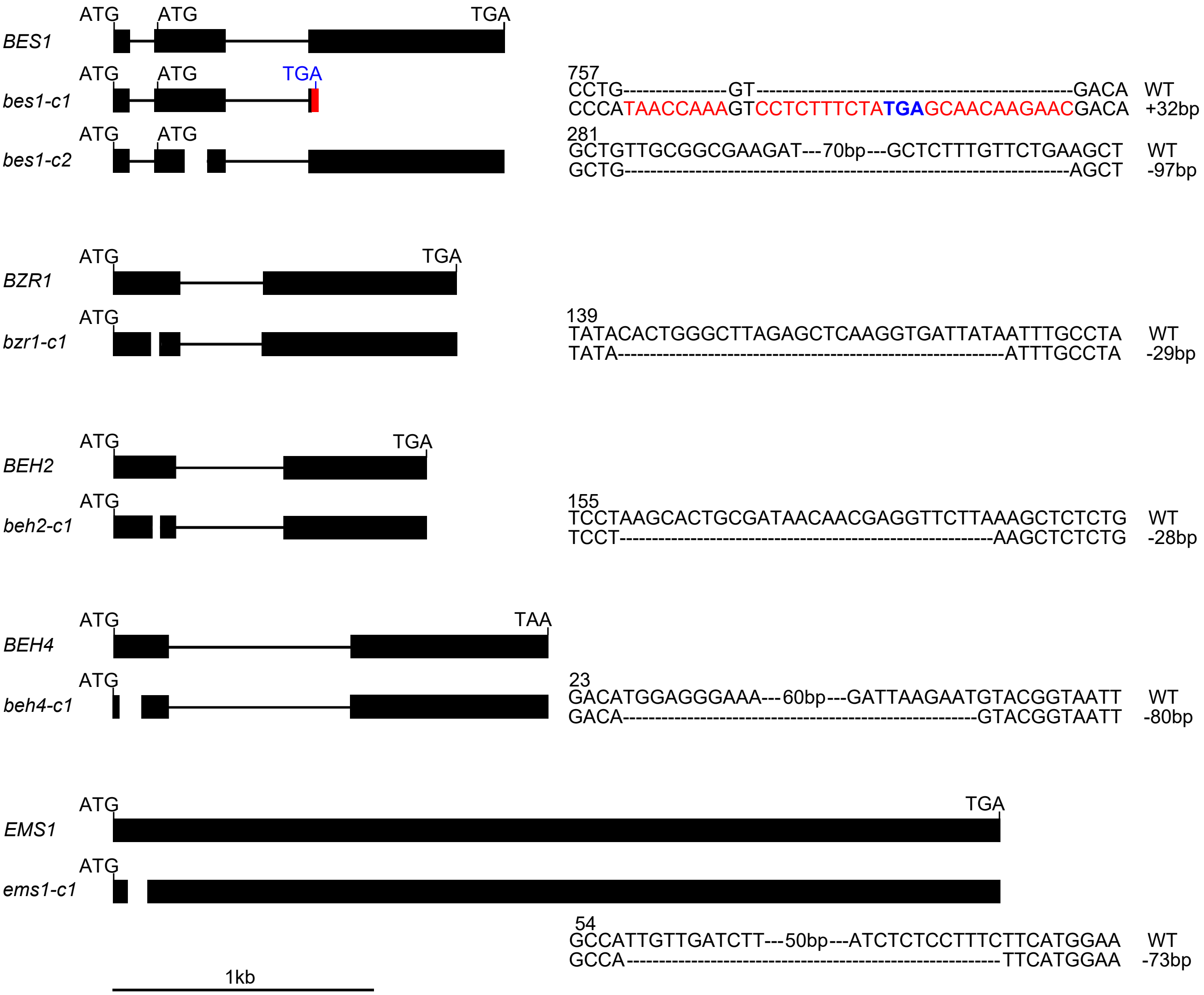

Supplementary Figure 7. Crispr-cas9 was used to generate edited lines of *bes1-c1*, *bes1-c2*, *bzr1-c1*, *beh2-c1*, *beh4-c1*, and *ems1-c1*. The deletion sites and deleted sequence for each crispr-cas9 mutant is shown in the diagram. Thick lines represent exons and thin lines represent introns. Red area represents the insertion of additional sequences from cripr-cas9 gene editing. Disrupted areas indicate deletion sites resulting from crispr-cas9 gene editing. Numbers above the DNA sequences represent the nucleotide position of the genomic DNA, starting from the initiation codon, ATG.

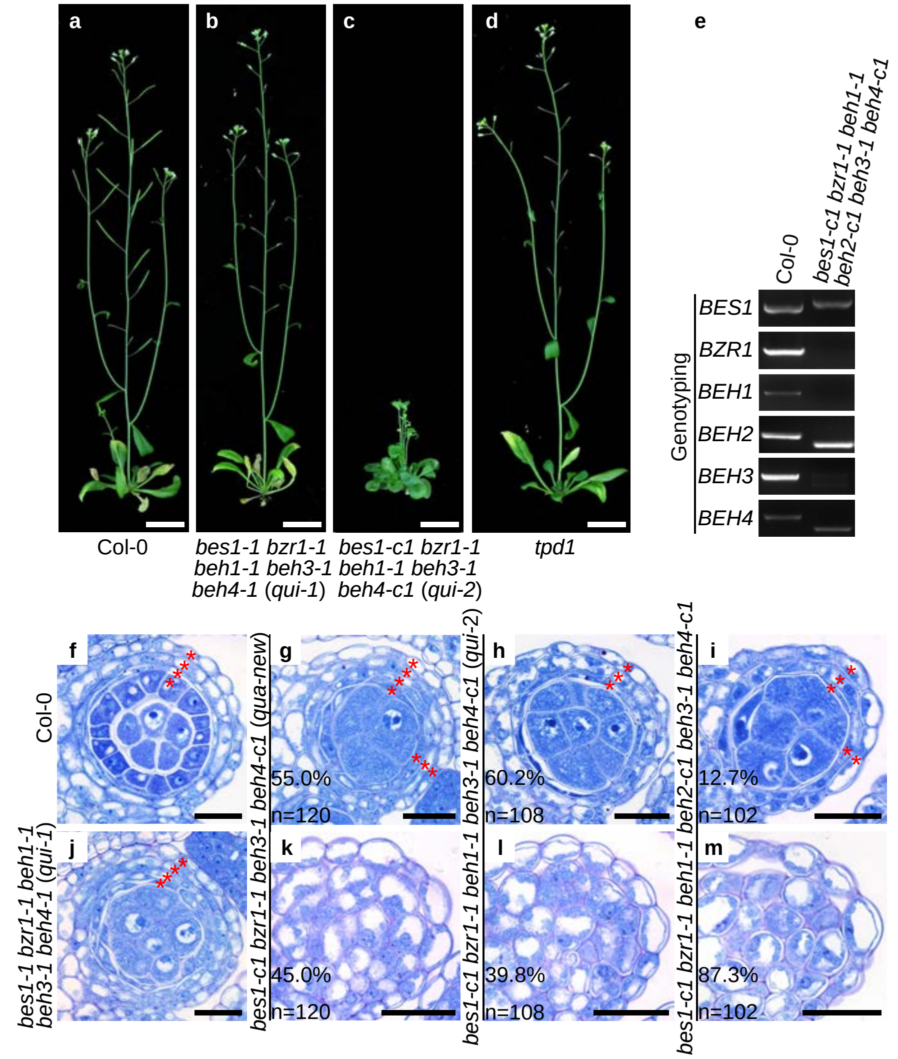

Supplementary Figure 8. Phenotypes and anther defects of various high order mutants from BES1 family. **a-d** Plant phenotypes of Col-0 (**a**), *qui-1* (**b**), *qui-2* (**c**), and *tpd1* (**d**). **e** PCR analyses to confirm the genotype of a sextuple mutant generated from the six BES1 family members. **f-m** Toluidine blue stained anther semithin sections to show anther phenotypes of Col-0 (**f**), *bes1-c1 bzr1-1 beh3-1 beh4-c1* (**g**, **k**), *bes1-c1 bzr1-1 beh1-1 beh3-1 beh4-c1* (*qui-2*) (**h**, **l**), *bes1-c1 bzr1-1 beh1-1 beh2-c1 beh3-1 beh4-c1* (**i**, **m**), and *bes1-1 bzr1-1 beh1-1, beh3-1 beh4-1* (*qui-1*) (**j**). Scale bars represent 2 cm in **a-d** and 20 nm in **f-m**.

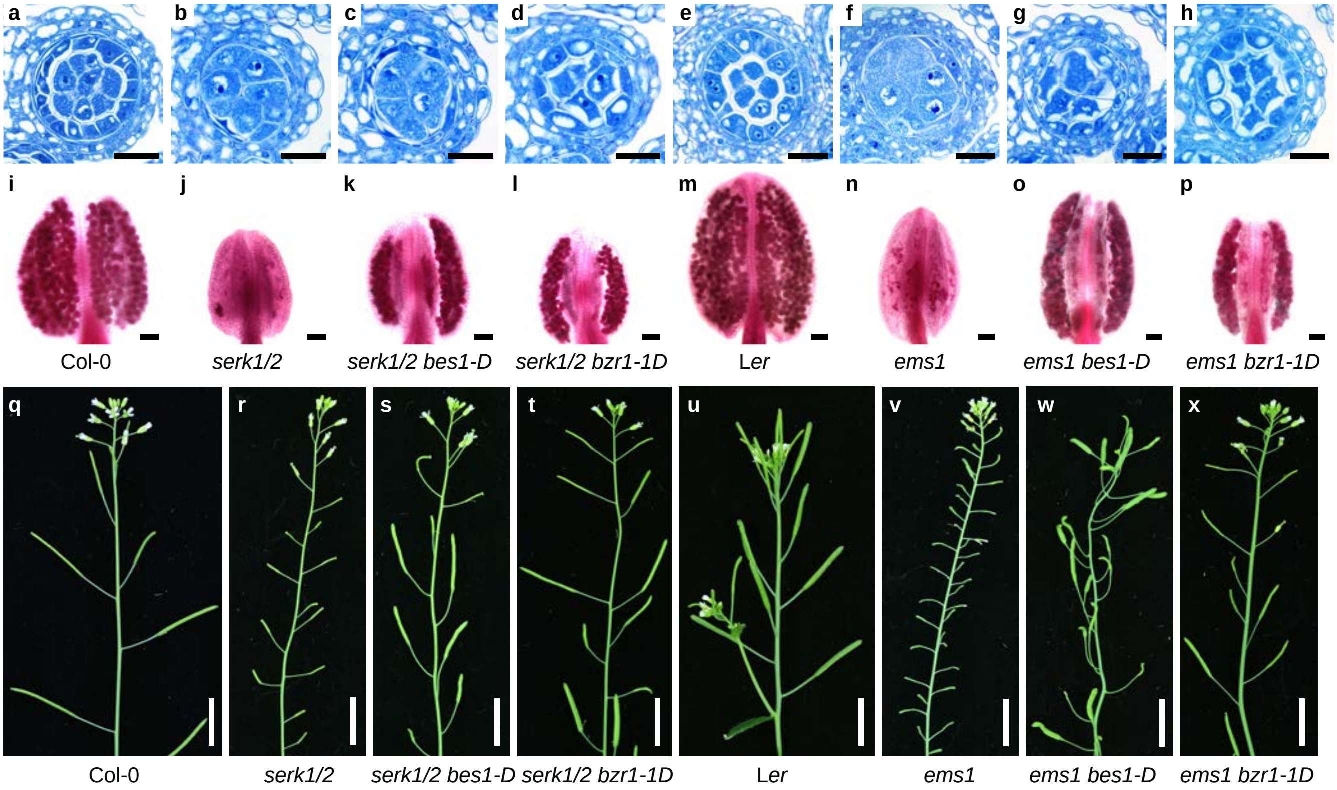

Supplementary Figure 9. *bes1-D* and *bzr1-1D* can partially suppress the anther and pollen developmental defects of *serk1 serk2* and *ems1*. **a-x** Toluidine blue stained semithin sections, Alexander stained anthers, and inflorescences of Col-0 (**a**, **i**, **q**), *serk1/2* (**b**, **j**, **r**), *serk1/2 bes1-D* (**c**, **k**, **s**), *serk1/2 bzr1-1D* (**d**, **l**, **t**), L*er* (**e**, **m**, **u**), *ems1* (**f**, **n**, **v**), *ems1 bes1-D* (**g**, **o**, **w**), and *ems1 bzr1-1D* (**h**, **p**, **x**). Scale bars represent 20 μm in **a-h**, 50 μm in **i-p**, and 1 cm in **q-x**.

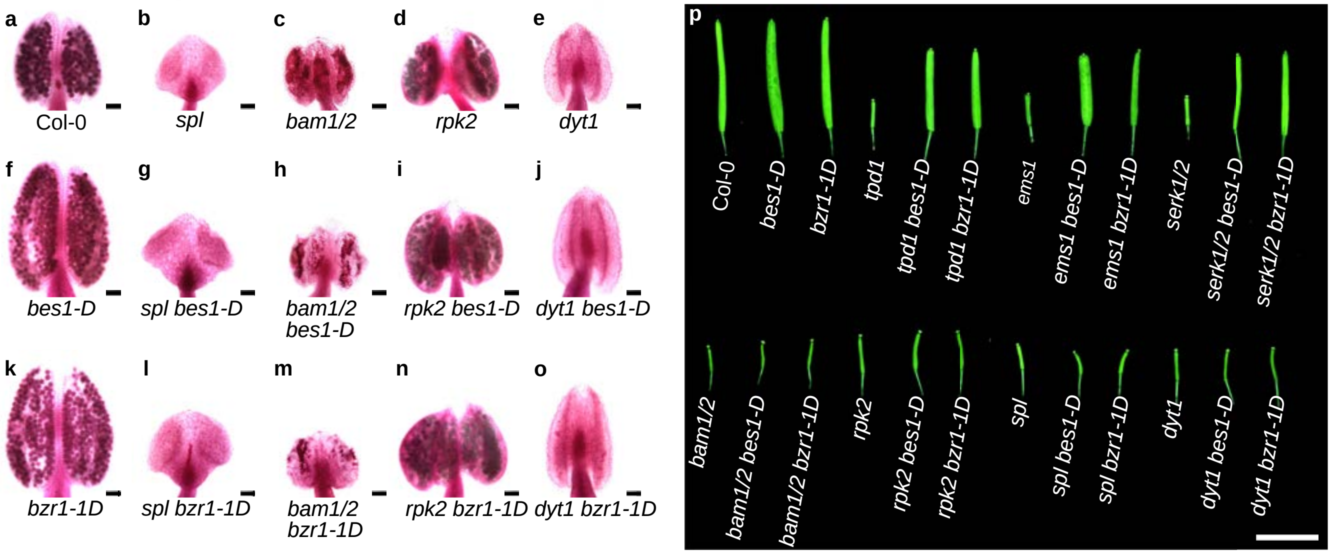

Supplementary Figure 10. *bes1-D* and *bzr1-1D* cannot suppress the male sterility phenotypes of *spl*, *bam1/2*, *rpk2*, and *dyt1*. **a-e** Alexander stained anther phenotypes of Col-0, *spl*, *bam1/2*, *rpk2*, and *dyt1*. **f-j** Alexander stained anther phenotypes of *bes1-D*, *spl bes1-D*, *bam1/2 bes1-D* , *rpk2 bes1-D*, and *dyt1 bes1-D*. **k-o** Alexander stained anther phenotypes of *bzr1-1D*, *spl bzr1-1D*, *bam1/2 bzr1-1D*, *rpk2 bzr1-1D* , and *dyt1 bzr1-1D*. (**p**) *bes1-D* and *bzr1-1D* can recover the fertility of *tpd1*, *ems1*, *serk1/2*, but cannot suppress the fertility of *bam1/2*, *rpk2*, *spl*, and *dyt1*. Scale bars represent 50 μm in **a-o** and 1 cm in **p**.

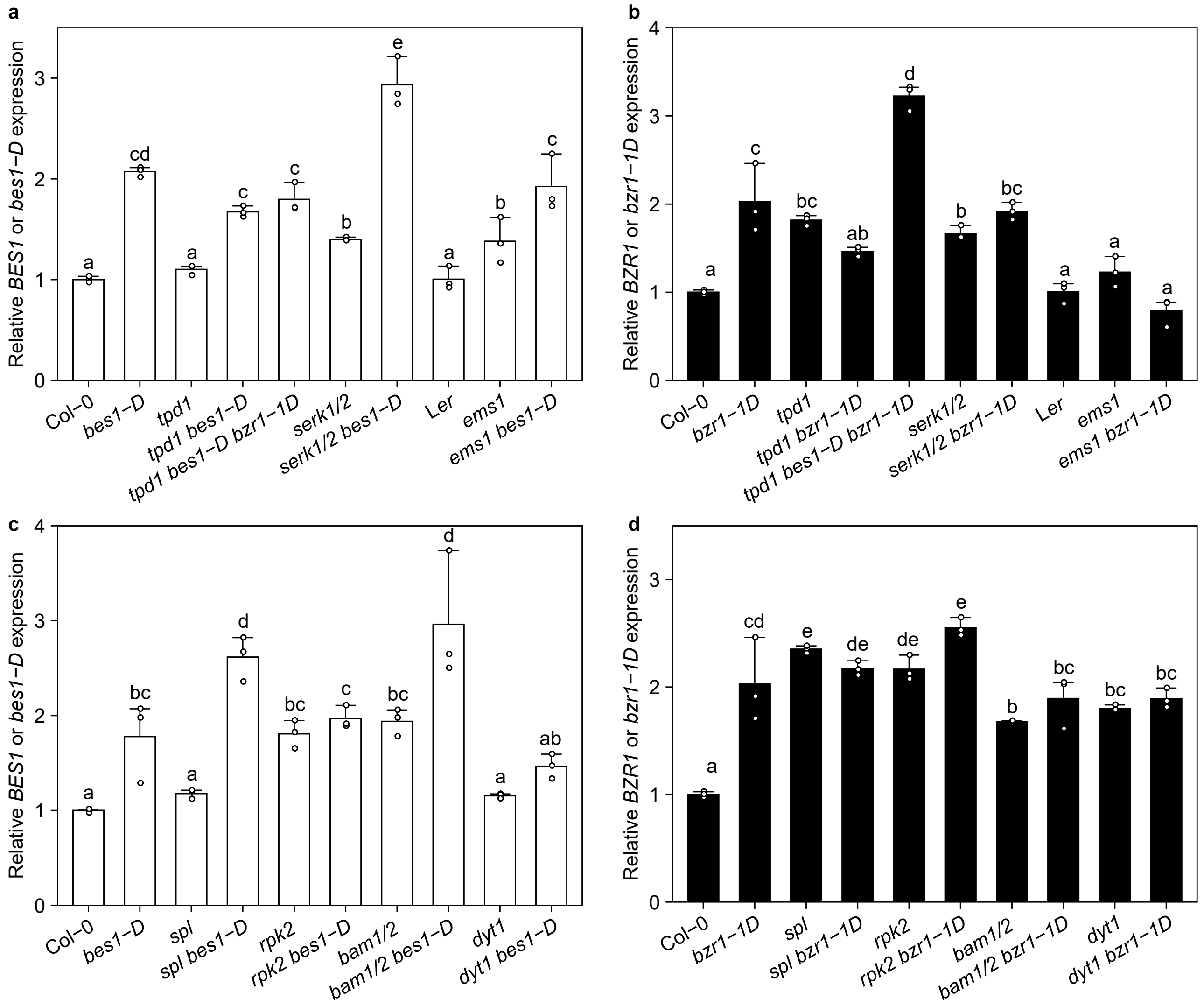

Supplementary Figure 11. Expression levels of *BES1/bes1-D* and *BZR1/bzr1-1D* in various indicated mutant backgrounds. Relative expression levels of *BES1/bes1-D* (**a**, **c**) and *BZR1/bzr1-1D* (**b**, **d**) were determined by quantitative RT-PCR analyses. *ACT2* was used as an internal control. Data are shown as mean and s.d. (n = 3). Statistically significant differences between groups were tested using One-way ANOVA followed by LSD (Least Significant Difference) post hoc test. Different letters above the bars indicate significant difference at $P < 0.05$.

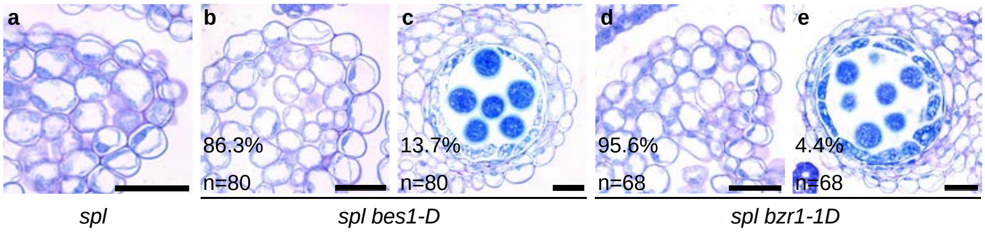

Supplementary Figure 12. Minor suppression of *spl* anther defect by *bes1-D* and *bzr1-1D*. Semi-thin cross sections of stage11 anthers were stained with Alexander solution. scale bar represent 20 μm.

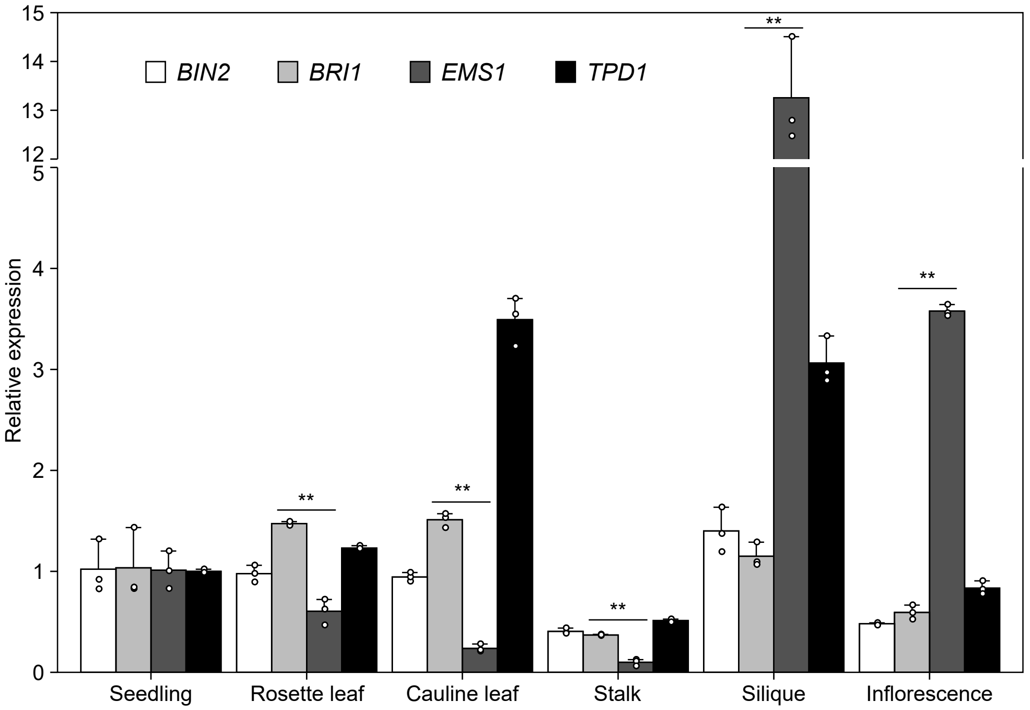

Supplementary Figure 13. Quantitative RT-PCR analysis to show differentiated expression of *BIN2*, *BRI1*, *EMS1*, and *TPD1* in several parts of Arabidopsis. Data are shown as mean and s.d. (n = 3). *ACT2* was used as an internal control. Asterisks indicate significant differences (P < 0.01, two-tailed t-test).

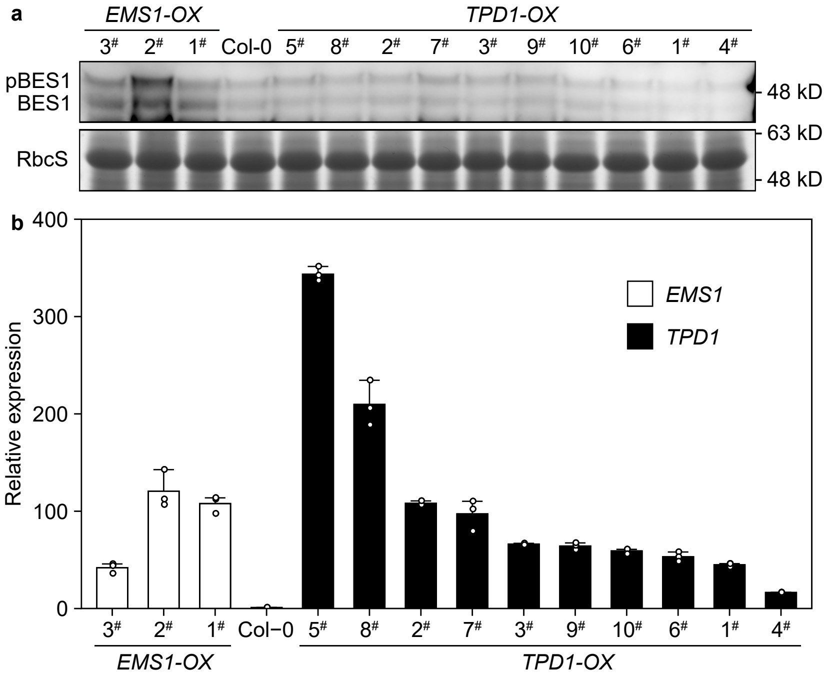

Supplementary Figure 14. Overexpression of *EMS1* but not *TPD1* alone can partially lead to the accumulation of non-phosphorylated BES1. **a** Immunoblotting assay using anti-BES1 antibody. The numbers above each lane represent independent transgenic lines. The total proteins were extracted from the cauline leaves of six-week-old Arabidopsis plants. Coomassie Brilliant Blue stained rubisco protein was used as loading controls. **b** Quantitative RT-PCR results to show relative expression levels of *EMS1* and *TPD1* in each corresponding individual transgenic plant. *ACT2* was used as an internal control. Data are presented as mean and s.d. (n = 3). Numbers at x axis represent individual transgenic lines.

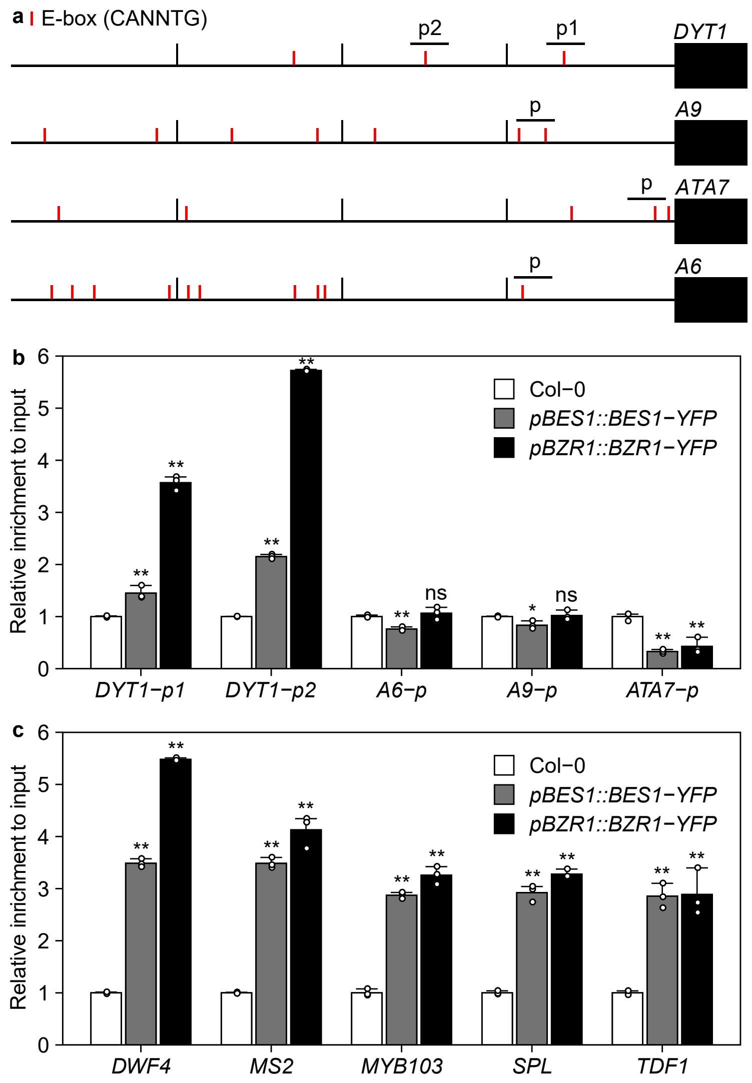

Supplementary Figure 15. ChIP analyses demonstrate BES1 and BZR1 can be enriched in the promoter regions of *DYT1*, but not in the promoter regions of *A6*, *A9*, and *ATA7*. **a** Schematic diagram showing the promoter regions of *DYT1*, *A6*, *A9*, and *ATA7*. Red upright lines represent E-box DNA motifs. The short black horizontal lines and p, p1, and p2 above indicate the DNA fragments for PCR amplification. **b**, **c** Quantitative RT-PCR assays to show the relative enrichment levels of indicated genes. *ACT2* was used as an internal control in PCR quantification. All relative enrichment levels were normalized against that in input controls. The enrichment of *DWF4*, *MS2*, *MYB103*, *SPL*, and *TDF1* were used as positive controls. Data are shown as mean and s.d. (n = 3). Asterisks indicate significant differences (**P < 0.01, *P < 0.05, two-tailed t-test).

Supplementary Table 1. Primers used in genotyping, cloning, RT-PCR, qRT-PCR, *in-situ* hybridization, and ChIP-qPCR

**For genotyping**.	
BES1-GT-F	AGGACTTAAACTTCGCTTTCAG
BES1-GT-R	ATGTCACCAGGTAGAGGCTT
BZR1-GT-F	CTGAAGAAACGGTCCCACT
BZR1-GT-R	TCTGAGGAAAGGGAAGAATGTA
BEH1-GT-F	ACCGAACATTGTCAGATGAA
BEH1-GT-R	TTTAAACGTGTATAAGGACAGAAG
BEH3-GT-F	TGTGGATAGTCCGTAGTTGG
BEH3-GT-R	TGTGTGAAAGCATCAGCGAATC
BEH4-GT-F	ACTGCAACTGTTTCCACGATTT
BEH4-GT-R	GAATCCAAATGGGTTTTGTGAG
**For cloning**.	
Ps1-s1-F	AAAAAGCAGGCTTCAATATAAATTCAATAAGTGACTACTCGTT
Ps1-s1-R	AGAAAGCTGGGTCACTATGAGCTTTACCATTTCCAAG
Pr1-r1-F	AAAAAGCAGGCTTCGATTGATGCTTTTAGAGGGTTTT
Pr1-r1-R	AGAAAGCTGGGTCACCACGAGCCTTCCCATTTCC
Ph1-h1-F	AAAAAGCAGGCTTCAATCGAAGAACCGAAGTATCAAT
Ph1-h1-R	AGAAAGCTGGGTCGCCGCGTCCTTTGGTGTTGTGA
Ph4-h4-F	AAAAAGCAGGCTTCCATTGTTGTAACGGCTTAGATGA
Ph4-h4-R	AGAAAGCTGGGTCCCTGGTGCTTGAGTTTCCAAGA
BEH3-C-F	AAAAAGCAGGCTTCATGACGTCGGGGACTAGAACG
BEH3-C-R	AGAAAGCTGGGTCTCTGGTTCTTGAGTTTCCAAGTGTAA
**For quantitative RT-PCR**.	
ACT2-Real-F	TGTGCCAATCTACGAGGGTTT
ACT2-Real-R	TTTCCCGCTCTGCTGTTGT
A6-Real-F	TACCTAAACCGACGAACA
A6-Real-R	ATGCCAATAAATGGAGAC
A9-Real-F1	TGCCCTCCAAGCAACTAACAA
A9-Real-R1	GCTGCTCGAAGAGCGTTACAT
ATA7-Real-F1	CGTCTCCAGGATCGAGGAAT
ATA7-Real-R1	GGAGATGGGAAAGCTGAGAG
MS1-Real-F	TGGTGGGTGGTCAAATAGAG
MS1-Real-R	TCATCATTCCTACGTTCCCT
AMS-Real-F	TCGTTGCTGGAAATAACCC
AMS-Real-R	TTTGCATAGAGCCTGTAGCC
TDF1-Real-F	CGGTTCCTCAAGTAGTGGG
TDF1-Real-R	ATGTATTCGGCTTCGATGTT
AtMYB103-F	AGATGGAATGACGATGATGAG
AtMYB103-R	GCTTGTAAATCCCACAAGACA
MS2-Real-F	GTATCAGATCGCTTCTTCGG

MS2-Real-R	TGCATGGGGATGTTTTGTA
DYT1-Real-F	TTATGAGATTTCTCGGATTCG
DYT1-Real-R	TCCTGTGTCTGAACAGAGGC
BES1-Real-F	CCAGCGAAGAAGAAAGAAAAAA
BES1-Real-R	GACGTCGACGTTGCTCCG
BZR1-Real-F	GAGGAGGAAGCCGTCGTG
BZR1-Real-R	AAGCCCAGTGTATATCTTCGCA
BEH1-Real-F	CGTCGGAGTAGATGTGTCGG
BRH1-Real-R	GCGTCCTTTGGTGTTGTGAC
BEH3-Real-F	GCTCCCTAAACACTGCGACA
BEH3-Real-R	TGCGGTAAGTAGTTCCGTCG
BEH4-Real-F	CGGGCTAGTGAAAGCATGGG
BEH4-Real-R	TGTGGCTTTGGAATTACCTGG
**For RT-PCR**.	
ACT2-RT-F	CAGTGGTCGTACAACCGGTATTG
ACT2-RT-R	TGCTGTGATTTCTTTGCTCATACG
BES1-S-RT-F	GATGACGTCTGACGGAGCAA
BES1-S-RT-R	GACACTGGTGGAGTGACAGG
BES1-L-RT-F	ATTCCAGCGAAGAAGAAAGA
BES1-RT-3UTR-R	CCAATCCTTCCTTCCGACAT
BES1-RT-CDSR	TCAACTATGAGCTTTACCATTTC
BZR1-RT-F	ATGACTTCGGATGGAGCTACGT
BZR1-RT-CDSR	TCAACCACGAGCCTTCCCATTT
BZR1-RT-3UTR-R	AAGATCCAAATACCTGGACGAAG
BEH1-RT-F	ATGACGGCATCAGGAGGAGGATC
BEH1-RT-CDSR	CTAGCCGCGTCCTTTGGTGTT
BEH1-RT-3UTR-R	TCGTACCGGACCCGACTAAA
BEH3-RT-F	ATGACGTCGGGGACTAGAACGC
BEH3-RT-CDSR	TCTGGTTCTTGAGTTTCCAAGTG
BEH3-RT-3UTR-R	CTAATCTCAAATCGAATTTCCAC
BEH4-RT-F	ATGACATCAGGGACGAGAATGC
BEH4-RT-CDSR	CCTGGTGCTTGAGTTTCCAAGA
BEH4-RT-3UTR-R	TTGGAGCAACATATCAAGAACG
TPD-F-CDS	ATGAACCGACGGCGACTTT
TPD-R-CDS	AGCACATGTCACGAAGGCG
**For *in-situ* hybridization.**	
DYT1-F	AAGCAGATTTCAAGAACCAGTG
DYT1-R	TCTCATAACTTCCAAAAGAAAATC
ATA7	TCACCTTCGCTAGCTAGTGTA
ATA7	GTTACAAGGCTTCCCTTC
**For ChIP-qPCR assay.**	
DYT1-p1-F	AGTTAGGGTTTCGTCGAGTCA
DYT1-p1-R	TCAAGGGTTCAAAACACGAATCC
DYT1-p2-F	AGACTGATCCATCCGGGGAA

DYT1-p2-R	CTTTGGCTCTTTCCGCGTTC
A6-p-F	ACTATGAGTGGTCGTCTAAAAGCC
A6-p-R	ATCTTCCGTGCACGAACCAAA
A9-p-F	AGTACAAGTGGTGGGAACGAA
A9-p-R	CCGGTTACACGTAAATGCTTGA
ATA7-p-F	GACACATCATACATGTCATGCGA
ATA7-p-R	TCTCATGTTTCCTTCTTACTCGGA
DWF4-p-F	CCCACTCTCGTCTCGTCATG
DWF4-p-R	CCAATGATTGCCGGAATG
MS2-p-F	TCCCAACACGAAGAAATGAG
MS2-p-R	TTGCCTTACGAATTCTTTCCA
TDF1-p-F	TCAACCTGAAGCAAGCGATA
TDF1-p-R	GATGCAATGCAAAAGCTCTG
SPL-p-F	CCTGCCTAAACCCAGCAATA
SPL-p-R	CAAGGAACTGGTAGGGCAAA
MYB103-p-F	CCTACCGGCTTAAGGGAATC
MYB103-p-R	TTGCAAAAGAAGAAAGGAAATG

Supplementary Table 2. Sequences of targets and primers used in crispr/Cas9

**Sequences of targets.**	
BES1-C1_ Targets 1	ATGATCCAGCCATGTCACC
BES1-C1_ Targets 2	TCCCGAGTCCTTCTCGAGT
BES1-C2_ Targets 1	CGGCGGAGAAGAGCTGTTG
BES1-C2_ Targets 2	AGGCTCTTTGTTCTGAAGC
BZR1-C1_ Targets 1	CGGCGGCAGCAGCGGCGAG
BZR1-C1_ Targets 2	TAGCTGCGAAGATATACAC
BEH2-C1_ Targets 1	CGTTGTTATCGCAGTGCTT
BEH2-C1_ Targets 2	GTTGGATCGTCGAAGACGA
BEH4-C1_ Targets 1	ACGAGAATGCCGACATGG
BEH4-C1_ Targets 2	TACATTCTTAATCCGGTGA
EMS1-C1_ Targets 1	GAGCTAAGATCAACAATGG
EMS1-C1_ Targets 2	CGAAACGTTCCATGAAGAA
**Primer used for cloning.**	
BES1DT1-BsF1	ATATATGGTCTCGATTGATGATCCAGCCATGTCACCGTT
BES1DT1-F01	TGATGATCCAGCCATGTCACCGTTTTAGAGCTAGAAATAGC
BES1DT2-R01	AACTCCCGAGTCCTTCTCGAGTCAATCTCTTAGTCGACTCTAC
BES1DT2-BsR1	ATTATTGGTCTCGAAACTCCCGAGTCCTTCTCGAGTC
BES1DT1-BsF2	ATATATGGTCTCGATTGCGGCGGAGAAGAGCTGTTGGTT
BES1DT1-F02	TGCGGCGGAGAAGAGCTGTTGGTTTTAGAGCTAGAAATAGC
BES1DT2-R02	AACGCTTCAGAACAAAGAGCCTCAATCTCTTAGTCGACTCTAC
BES1DT2-BsR2	ATTATTGGTCTCGAAACGCTTCAGAACAAAGAGCCTC
BZR1DT1-BsF1	ATATATGGTCTCGATTGCGGCGGCAGCAGCGGCGAGGTT
BZR1DT1-F01	TGCGGCGGCAGCAGCGGCGAGGTTTTAGAGCTAGAAATAGC
BZR1DT2-R01	AACGTGTATATCTTCGCAGCTACAATCTCTTAGTCGACTCTAC
BZR1DT2-BsR1	ATTATTGGTCTCGAAACGTGTATATCTTCGCAGCTAC
BEH2DT1-BsF1	ATATATGGTCTCGATTGCGTTGTTATCGCAGTGCTTGTT
BEH2DT1-F01	TGCGTTGTTATCGCAGTGCTTGTTTTAGAGCTAGAAATAGC
BEH2DT2-R01	AACGTTGGATCGTCGAAGACGACAATCTCTTAGTCGACTCTAC
BEH2DT2-BsR1	ATTATTGGTCTCGAAACGTTGGATCGTCGAAGACGAC
BEH4DT1-BsF1	ATATATGGTCTCGATTGGACGAGAATGCCGACATGGGTT
BEH4DT1-F01	TGGACGAGAATGCCGACATGGGTTTTAGAGCTAGAAATAGC
BEH4DT2-R01	AACTACATTCTTAATCCGGTGACAATCTCTTAGTCGACTCTAC
BEH4DT2-BsR1	ATTATTGGTCTCGAAACTACATTCTTAATCCGGTGAC
EMSDT1-BsF1	ATATATGGTCTCGATTGGAGCTAAGATCAACAATGGGTT
EMSDT1-F01	TGGAGCTAAGATCAACAATGGGTTTTAGAGCTAGAAATAGC
EMSDT2-R01	AACTTCTTCATGGAACGTTTCGCAATCTCTTAGTCGACTCTAC
EMSDT2-BsR1	ATTATTGGTCTCGAAACTTCTTCATGGAACGTTTCGC
**Primers used for genotyping.**	
BES1-CRSGT-F1	GAGAAAGTGACCTTTAGGGTTC
BES1-CRSGT-R1	CCCAAGTAGGCAATGGTTT

BES1-CRSGT-F2	AGGAGGAAACCGTCGTGG
BES1-CRSGT-R2	TCTCCGCAAGGAATCTCG
BZR1-CRSGT-F1	CACAATCTTTCACCCACCTAT
BZR1-CRSGT-R1	TTCAAACCCATTTAACCACAA
BEH2-CRSGT-F1	GCTCAGATTCCGATCTAAAA
BEH2-CRSGT-R1	AGAAGAGCCATCACTGCTAA
BEH4-CRSGT-F1	GTTGGTTCACGCAGTATCG
BEH4-CRSGT-R1	TAAGTAGTTCCATCAGGTTCG
EMS1-CRSGT-F1	TCTTACCGCATTGTTCCTGT
EMS1-CRSGT-R1	TGGCCTCTCAGAGACAAAGA
